# Preparation of diethylene glycol monomethyl ether monolaurate catalyzed by active carbon supported KF/CaO

**DOI:** 10.1186/s40064-015-1486-5

**Published:** 2015-11-10

**Authors:** Shengfeng Lou, Lihua Jia, Xiangfeng Guo, Ping Wu, Lianbing Gao, Jianjun Wang

**Affiliations:** College of Chemistry and Chemical Engineering, Key Laboratory of Fine Chemicals of College of Heilongjiang Province, Qiqihar University, Qiqihaer, 161006 China; Heilongjiang Haohua Chemical Corporation Limited, Qiqihaer, 161033 China

**Keywords:** Diethylene glycol monomethyl ether monolaurate, KF, CaO, Active carbon, Transesterification

## Abstract

Diethylene glycol monomethyl ether monolaurate (DGMEML) was synthesized via the reaction of diethylene glycol monomethyl ether (DGME) with methyl laurate (ML) by a new solid base catalyst of KF/CaO/AC, which was prepared by impregnation method using active carbon as carrier. The catalysts were characterized by X-ray diffraction (XRD), Fourier transform infrared spectroscopy (FT-IR), scanning electron microscopy (SEM), nitrogen physisorption-desorption and Hammett indicator methods; the effect of the mole ratio of KF to CaO, DGME to ML molar ratio, amount of catalyst, reaction time and temperature on the yield of DGMEML were studied; and the relationship between the structure of the catalyst and the yield of DGMEML was investigated. The formed KCaF_3_ and K_2_O were acting as the main active components in the catalytic transesterification; the highest yield of 96.3 % was obtained as KF-to-CaO molar ratio of 2.0, DGME to ML molar ratio of 4.0, catalyst amount of 5 wt%, and reaction time of 30 min at 75 °C; and the catalyst displayed good stability in the transesterification.

**Electronic supplementary material:**

The online version of this article (doi:10.1186/s40064-015-1486-5) contains supplementary material, which is available to authorized users.

## Background

Biodiesel originated from the transesterification of vegetable oils or animal fats with short chain alcohols has been attracted more attention for the biodegradable, nontoxic, comparable calorific value and relative lower emission of NO_*x*_ and CO_2_ to the petroleum diesel; and the production of biodiesel increased rapidly around the world (Shahir et al. 2015; Avhad and Marchetti 2015; Gopinath et al. 2015). In recent years, some studies tried successfully to introduce one ether group or more into biodiesel molecules to further reduce smoke emissions, and a novel biodiesel synthesized via the transesterification of fatty acids methyl esters with short chain glycol ethers, such as ethylene glycol monobutyl ether palm oil monoester (Jiang and Yun 2012), ethylene glycol *n*-propyl ether palm oil monoester (Gao et al. 2011), ethylene glycol monoethyl ether soybean oil monoester (Zhang et al. 2006), and ethylene glycol monomethyl ether palm oil monoester (Jiang et al. 2011; Guo et al. 2015a) et al. have been developed. Compared with the traditional biodiesel, novel biodiesel with higher oxygen content for introducing an ether group can effectively improve the combustion and emission performance (Guo et al. 2013; Guo et al. 2015b; Chen et al. 2014a). At present, novel biodiesel is mainly prepared by homogeneous base catalyst, such as sodium alcoholate (Zhang et al. 2012; Guo et al. 2015c) and KOH (Li et al. 2012; Jiang 2012). The usage of homogeneous catalysts produced large amounts of caustic wastewater giving rise to serious environmental pollution and post-processing was complex (Deshmane and Adewuyi 2013). Recently, Na_2_SiO_3_ (Fan et al. 2013), KF/HTL (Chen et al. 2014b) and KF/CaO/Kaolinite (Guo et al. 2015c) acting as solid base catalysts have been respectively used in the production of novel biodiesel of ethylene glycol monomethyl ether soybean oil ester and ethylene glycol monomethyl ether monolaurate. The results demonstrates the heterogeneous solid bases show good catalytic performance, and the catalytic processes have fewer unit operations. Moreover, the simple methods of filtration, centrifugation can be easily used to separate the solid catalyst from the reaction system. It has becoming a promising route for the production of novel biodiesel.

Diethylene glycol monomethyl ether-based biodiesel which contains two ether groups have higher oxygen content. It was found that the density, kinematic viscosity, smoke point, and cetane number of diethylene glycol monomethyl ether-based biodiesel increased obviously compared with that of traditional biodiesel and ethylene glycol monomethyl ether-based biodiesel (Guo et al. 2013, 2015a). Few reports about the solid base catalyst used in production of diethylene glycol monomethyl ether-based biodiesel via diethylene glycol monomethyl ether with fatty acid methyl ester has been revealed.

KF/CaO catalysts showed higher catalytic activity in the manufacture of biodiesel, but it is not easy to separate (Hu et al. 2012; Fan et al. 2014; Jia et al. 2015). Activated carbon with a large surface area as supporter is widely used in the production of biodiesel for the dispersion of active sites effectively, and the surface characteristic of activated carbon does not change at high temperature or pressure (Naranjo et al. 2010; Baroutian et al. 2010; Buasri et al. 2012; Li et al. 2013; Malins et al. 2015; Tao et al. 2015). Inspired by the previous reports, an effective and separable solid bases of active carbon supported KF/CaO was prepared by impregnation method, and tried to use as a catalyst in the transesterification of diethylene glycol monomethyl ether (DGME) and methyl laurate (ML) to produce diethylene glycol monomethyl ether monolaurate (DGMEML). X-ray diffraction (XRD), Fourier transform infrared spectroscopy (FT-IR), scanning electron microscopy (SEM), Hammett indicator and nitrogen physisorption-desorption were performed to characterize the structure of the catalysts, in an attempt to explain the correlation between structure and activity of the catalyst. In addition, the effect of mole ratio of KF to CaO and main reaction parameters on the yield of DGMEML was investigated. The catalyst showed an excellent catalytic activity, and could be easily to separate from the system.

## Methods

### Materials and methods

All of the chemicals used in the present work were of A.R. grade and purchased from Aladdin, China. DGMEML sample was synthesized by our laboratory reference to the literature (Jiang 2012), and the structure was confirmed by ^1^H NMR, ^13^C NMR and FT-IR spectrum, which are shown in SI.

The active carbon-based catalyst was prepared by wet impregnation method. In order to remove any soluble alkaline and acidic impurities from the AC, a pretreatment was first performed by washing the support with 0.1 M NaOH solution, followed by the second treatment with 0.1 M HCl (Badday et al. 2014). Available active carbon obtained after drying at 80 °C for 24 h. 0.56 g (0.01 mol) CaO powder and 1.00 g active carbon were immersed in 10 mL distilled water, and stirred for 4 h at 80 °C. After the water was evaporated, the solid CaO-active carbon was immersed in KF·2H_2_O solution with different amounts of KF for 1 h, and then stirred for 4 h at 80 °C, and followed by calcination in muffle furnace at 500 °C for 5 h. The prepared catalyst was sealed by plastic membrane for further use. The resultant composites were named as KCC-*n*, where ‘*n*’ stands for the molar ratio of KF to CaO.

### Catalysts’ characterization

The basic strength of the sample (*H*_-_) was determined using Hammett indicator. About 50.0 mg of the sample was shaken with 5.00 mL cyclohexane and two or three drops of Hammett indicators-benzene solution (0.1 %, w/w) and then left to equilibrate for 2 h when no further color changes were observed. The Hammett indicators used and the corresponding *H*_−_ values are as follows: 4-nitroaniline (*H*_−_ = 18.4), 2, 4-dinitroaniline (*H*_−_ = 15.0), phenolphthalein (*H*_−_ = 9.8) (Boz et al. 2009; Yan et al. 2009). Alkaline determination was measured by benzoic acid titration method, using 0.02 mol/L benzoic acid-anhydrous ethanol solution as titrant, until the basic color of indicator adsorbed on the surface of solid alkali just disappeared. Powder XRD diffraction was recorded on a Bruker D8 Advance (Germany) diffractometer, using Cu Kα radiation (λ = 1.5418° A) at 40 kV and 50 mA. The scanning speed was 3° min^−1^ and scanned area ranged 2θ = 10–80°. Morphology of the samples was observed by SEM using a Rigaku S-4700 spectrometer (Japan). The voltage was 20 kV and the vacuum degree of the sample room was better than 10^−4^. The BET surface areas and pore sizes were measured using a NOVA 2000e operating on the basis of the physical adsorption of liquid nitrogen performed at a temperature of 77 K by the Brunauer-Emmett-Teller (BET) method. FT-IR spectra were recorded on an AVATAR370 FT-IR spectrophotometer both over the wave number range from 400 to 4000 cm^−1^ at a resolution of 4 cm^−1^ and by averaging over 16 scans. Samples were prepared by mixing the powdered solids with potassium bromide.

### Transesterification reaction

**Scheme 1:**
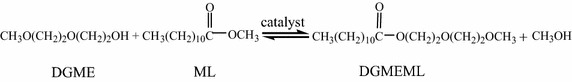
Transesterification process to produce DGMEML

KCC-*n* was used as catalyst in transesterication between methyl laurate (ML) and diethylene glycol monomethyl ether (DGME) (Scheme 1).

4.28 g (20.0 mmol) of ML, desired amount of DGME and as-developed catalyst mixed in a glass flask, equipped with a thermometer, and a water segregator connected with water-cooled condenser. The reaction was kept in 0.015 MPa with vacuum pump. At the end of the reaction, quickly stopped by cooling and separated the catalyst from the reaction system by decantation and centrifugation. The excess DGME and byproduct methanol of supernatant liquid was removed by a rotary vacuum evaporator. The residue was quantitatively analyzed by gas chromatography (GC) based on peak area normalization method in a SP-6890 (China) equipped with a fame ionization detector and an OV-101 capillary column (30 m × 0.25 mm × 0.25 μm). Initially, the oven temperature was kept at 180 °C for 1 min; then, it was gradually increased at 10 °C min^−1^ up to 220 °C and held for 0.5 min, and ramped at 10 °C min^−1^ up to 280 °C, the detector temperature was kept at 280 °C throughout the analysis. The injector and detector temperatures were both 300 °C. The yield (Y) was calculated by the following equation (Fan et al. 2013; Chen et al. 2014a):

$$Y = \frac{{m_{1} \times w}}{{m_{2} }} \times 100\;{\text{\% }}$$

where *m*_*1*_ is the actual mass of biodiesel (g), *m*_*2*_ is the theoretical mass of the biodiesel (g), and *w* is the mass concentration of the novel biodiesel determined by GC.

## Results and discussion

### Catalyst characterization

**Fig. 1:**
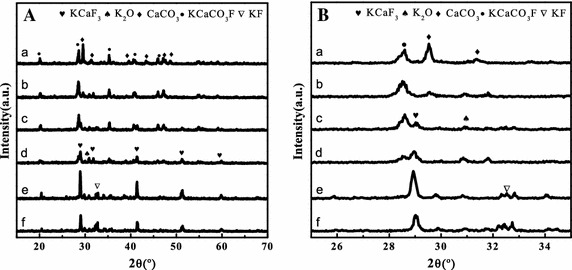
The XRD patterns (**A**) and their enlarged view (**B**) of KCC catalysts.KCC-0.5 (*a*); KCC-1.0 (*b*); KCC-1.5 (*c*); KCC-2.0 (*d*); KCC-2.5 (*e*); KCC-3.0 (*f*)

XRD patterns of KCC catalysts with different KF-to-CaO molar ratio are shown in Fig. 1. As *n* increased from 0.5 to 3.0, the characteristic peaks intensity of the dominant compounds KCaCO_3_F (20°, 29°, 35°, 41°, 45°, 55°, 58°) (Gao et al. 2010) and CaCO_3_ (23.3°, 29.6°, 31.5°, 36.2°, 39.7°, 43.4°, 47.8°, 48.8°) (Sun et al. 2014) became weaker; and the new phases at 28.8°, 41.2°, 51.2°, 59.7° and 31° appeared, which respectively belongs to KCaF_3_ and K_2_O (Isahak et al. 2012). KCaF_3_ and K_2_O generated from KF interacted with CaO. However, all of these XRD spectrums did not show any peaks of CaO, this might be the particles on the active carbon were so small that there high surface made it could reacted easily, and the CaO interacted with water and CO_2_ of air and formed CaCO_3_ with contraction of air. When *n* exceeds 2.0, the signal at 33.5^o^ appeared due to the excess of KF (Xuan et al. 2012). It may be that the capacity for KF dispersion on catalyst reached the limit. When KF loading exceeds the dispersion capacity, there is blocking of active sites and agglomeration of crystallites (Wei et al. 2011).

**Fig. 2:**
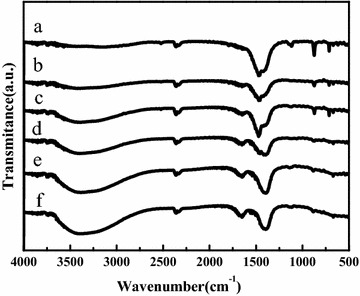
FT-IR patterns of the catalysts. KCC-0.5 (*a*); KCC-1.0 (*b*); KCC-1.5 (*c*); KCC-2.0 (*d*); KCC-2.5 (*e*); KCC-3.0 (*f*)

Figure 2 depicts the FT-IR patterns of KCC catalysts with different molar ratio of KF-to-CaO. The absorption peaks at 700–1500 cm^−1^ belongs to different forms of CO_3_^2−^ vibration peaks (Yamaguchi et al. 2002; Alonso et al. 2007), and the peak intensity of 1384 cm^−1^ corresponds to carbonate increased (Zeng et al. 2008), it was probably due to the base sites of KCC absorbed CO_2_ from the air. New absorption peaks appeared at 1640 cm^−1^ and 3200 cm^−1^ which could be ascribed to the *δ*_OH_ bending vibration mode of H_2_O molecules absorbed from air and the *ν*_OH_ stretching vibration of the hydroxyl groups respectively (Xie and Li 2006), and the intensity of the bands became stronger with the increase of KF mass. It may resulted from the base sites of KCC absorbed water molecules from the atmosphere, so the vibration resulted from the OH stretching vibration aggrandized with the increase of base sites.

**Fig. 3:**
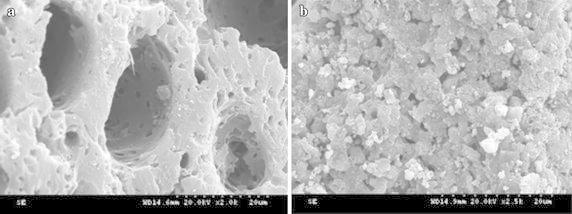
SEM images of active carbon (**a**) and KCC-2.0 (**b**)

SEM was employed to investigate the morphologies of the samples. The carrier of active carbon was with obvious pore structure (Fig. 3a), after loading KF/CaO, the pore structure of activate carbon was not destroyed, the active component evenly dispersed on the surface and in the hole of catalyst as shown in Fig. 3b, which is favorable for the catalytic reaction.

Active carbon is neutral, however, the synthesized KCC could change the color of phenolphthalein (*H*_-_ = 9.8) from colorless to purple, but failed to convert 2,4-dinitroaniline (*H*_-_ = 15.0) from yellow to mauve, and therefore, their basic strength could be tentatively denoted as 9.8 < *H*_-_ < 15.0, which was same as that of report (Liu et al. 2012). As shown in Table 1, basic strength and basicity of KCC improved when ‘*n*’ increased from 0.5 to 2.5, while the basicity and basic strength slightly change with *n* = 3.0, which is similar with that of the cinder supported K_2_CO_3_ (Liu et al. 2011). Surface area of KF/CaO supported on activated carbon was also studied. The significant reduction in BET surface area from activated carbon (761 m^2^/g) to the KF/CaO/AC catalyst (149.5 m^2^/g) indicates filling of active groups molecules into the activated carbon pores and confirmed the results of SEM. In conclusion, KCC is a strong basic catalyst, and it could be use in transesterifcation reaction.

**Table 1:** Basic strength and basicity of KCC-*n* catalysts

Catalyst	Basic strength (H_-_)	Basicity (mmol/g)	Yield (%)^a^
KCC-0.5	7.2 < *H*_−_ < 9.8	0.32	23.0
KCC-1.0	7.2 < *H*_−_ < 9.8	1.36	68.2
KCC-1.5	9.8 < *H*_−_ < 15.0	1.82	85.6
KCC-2.0	9.8 < *H*_−_ < 15.0	2.24	96.3
KCC-2.5	9.8 < *H*_−_ < 15.0	2.28	97.0
KCC-3.0	9.8 < *H*_−_ < 15.0	2.30	97.3

^a^Reaction condition: the amount of catalyst 5.0 %,reaction time 30 min, molar ratio of DGME/ML of 4.0, reaction temperature of 75 °C

### Catalytic activity of KCC

#### Effect of KF-to-CaO molar ratio

The effect of KF-to-CaO molar ratio of KCC-*n* on catalytic performance was investigated as shown in Table 1. With the increase of *n* from 0.5 to 2.0, DGMEML yield increased from 23.0 to 96.3 %. The catalytic activity increased significantly which lined with the basicity of the catalysts. Combined with the XRD patterns, the raising of DGMEML yield could be mainly related to the amount increase of KCaF_3_ and K_2_O phases. However, further increased *n* to 3.0, led to the less increase in the resulting basicity of the composite and thus caused a slightly increase in the DGMEML yield as seen in Table 1. Similar phenomenon was observed in literature (Algoufi et al. 2014). On the basis of the results, 2.0 was chosen as the optimum KF-to-CaO molar ratio.

#### Infuence of the reaction parameters

**Fig. 4:**
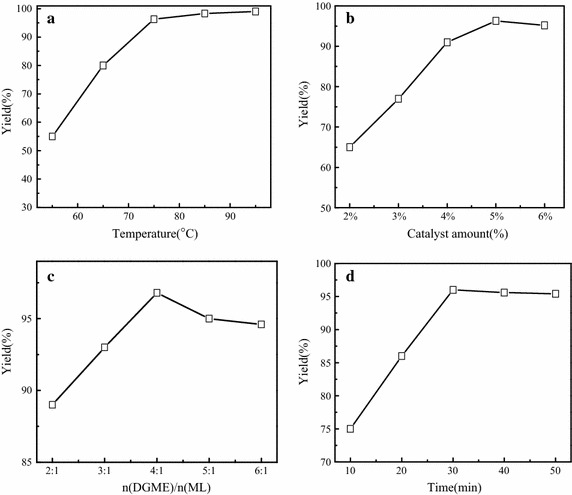
Influence of reaction condition on the yield of DGMEML. Reaction condition: the amount of catalyst 5.0 %,reaction time 30 min, molar ratio of DGME/ML of 4.0 (**a**); molar ratio of DGME/ML of 4.0, reaction time of 30 min, and reaction temperature of 75 °C (**b**); the amount of catalyst 5.0 %, reaction time of 30 min, and reaction temperature of 75 °C (**c**); molar ratio of DGME/ML of 4.0, amount of catalyst of 5.0 wt% and reaction temperature of 75 °C (**d**)

The transesterifcation reaction could be mainly influenced by the following parameters, such as DGME/ML molar ratio, amount of catalyst, reaction time and temperature. The effect of those parameters on the yield of DGMEML was examined as shown in Fig. 4. With the increase of temperature from 55 to 75 °C, the yield of DGMEML increased obviously from 55 to 96 % in Fig. 4a. However, the yield increased slightly with the increase of temperature above 75 °C. The increase of temperature can not only speed up the reaction rate, but also improve the miscibility between the reactants. But taking into account energy consumption, the optimal temperature of 75 °C was selected to be the most appropriate. Catalyst amount is an important parameter which affects the yield of DGMEML. Figure 4b showed that the yield increased from 65.5 % to a maximum of 96 % when the catalyst amount increased from 2 to 5 %, with further increase of KCC-2.0 amount, the yield decreased, which was possibly due to a mixing problem involving reactants, products and solid catalyst (Xie et al. 2006). It can also raise the cost of biodiesel production. The highest DGMEML yield was observed by using the 5 % catalyst. In addition, the effect of different molar ratio of DGME/ML on the yield of DGMEML was also studied (Fig. 4c). The yield increased from 88.4 % to a maximum of 96.3 % when the molar ratio of DGME/ML was increased from 2:1 to 4:1. However, the yield did not vary much with an increasing molar ratio of DGME/ML from 5:1 to 6:1. Due to the reversibility of reaction, a higher molar ratio is required to get desired yield, but will increase cost for excess alcohol recovery (Wan et al. 2014). And the excess of DGME may dilute the reaction system, resulted in the reaction rate reduction (Song et al. 2011). Thus, the molar ratio of DGME/ML was suggested to be 4:1. Reaction time between 10 and 50 min on DGMEML yield was analyzed and obtained data presented in Fig. 4d. The yield of the DGMEML increased from 75 to 96 % with the time going by until 30 min. After 30 min, the yield was not significantly increased. It indicates that the reaction reached the equilibrium state. Considering the problem of energy, 30 min is enough for the reaction time.

According to the above-mentioned experiments, the high yield of DGMEML was obtained under DGME/ML molar ratio of 4.0, catalyst amount of 5 wt% with respect to ML, reaction duration of 30 min at 75 °C.

#### Production of novel biodiesel of diethylene glycol monomethyl ether soybean oil monoester

Based on the optimized conditions of the transesterification of ML with DGME, KCC-2.0 was used as a catalyst in the production of a novel biodiesel of diethylene glycol monomethyl ether soybean oil monoester from the raw material of methyl soybean oil ester biodiesel and DGME (Additional file 1). As expected, the novel biodiesel yield of 90.0 % was obtained when the soybean biodiesel/DGME molar ratio was 1:8, the reaction temperature was 75 °C, the mass amount of catalyst was 5 %, and the reaction time was 30 min. It can be concluded that KCC-2.0 is an efficacious catalyst for the synthetic of soybean oil-base novel biodiesel.

#### The stability of the catalyst

**Fig. 5:**
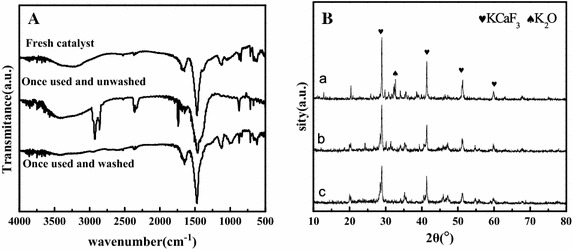
FT-IR (**A**) and XRD (**B**) patterns of fresh catalyst (*a*), catalyst used and unwashed (*b*) and catalyst used and washed (*c*)

After transesterification under the optimal conditions, centrifugation and decantation was successively used to separate the catalyst from the mixture, and the catalyst was kept in the flask and directly used in the next round of reactions. It is noted that DGMEML yield of 80 % were obtained as the KCC-2.0 used in the next reaction. While KCC-2.0 used once and washed by acetone, the yield of DGMEML was 89 %. It can be found from Fig. 5A, all the characteristic absorption bands of the used catalyst are the same with that of fresh catalyst. However, unwashed catalyst appeared three new bands, the new peaks at 2850 and 2920 cm^−1^ attributed to the present of C-H stretching vibrations(Li et al. 2015), and absorption peak at 1740 cm^−1^ belongs to the C=O stretching vibration. After the catalyst washed by acetone, the absorption peaks at 2920, 2860 and 1740 cm^−1^ disappeared. It indicates the surface of the used catalyst was covered by the organic oligomer produced in the reaction process, which resulted in the decrease of the activity of the used KCC-2.0. XRD patterns of used KCC catalysts are shown in Fig. 5B. It can be seen that the intensity of KCaF_3_ characteristic peaks decreased when the catalyst used and washed by acetone. The characteristic peak of K_2_O was disappeared. So, the decrease of catalytic activity may due to the loss of the active compound on the surface, which may run off during the recycle process.

## Conclusions

Diethylene glycol monomethyl ether monolaurate (DGMEML) was successfully synthesized via the transesterification of diethylene glycol monomethyl ether (DGME) with methyl laurate (ML) by an effective solid base catalyst of active carbon supported KF/CaO. Analyses by various modern instruments revealed that K_2_O and KCaF_3_ were active groups. The highest yield of DGMEML of 96.3 % was obtained as KF/CaO molar ratio of 2.0, DGME/ML molar ratio of 4.0, catalyst amount of 5 wt%, and reaction time of 30 min at 75 °C; and the yield of DGMEML was satisfied as the catalyst used in the next round. Furthermore, a desirable yield of 90.0 % of novel biodiesel of diethylene glycol methyl ether soybean oil monoester was obtained with KCC-2.0 as catalyst.

## Additional file

10.1186/s40064-015-1486-5 Detailed procedure of diethylene glycol monomethyl ether soybean oil monoester syntheis.
