# Supplementary material for: Preparation of diethylene glycol monomethyl ether monolaurate catalyzed by active carbon supported KF/CaO
Source: Springerplus. 2015 Nov 10;4:686. doi: 10.1186/s40064-015-1486-5 (PMC4641147; doi:10.1186/s40064-015-1486-5)
Supplement: Supplementary file 1 — 10.1186/s40064-015-1486-5 Detailed procedure of diethylene glycol monomethyl ether soybean oil monoester syntheis. [file 40064_2015_1486_MOESM1_ESM.doc]

Preparation of Diethylene glycol monomethyl ether monolaurate catalyzed by active carbon supported KF/CaO


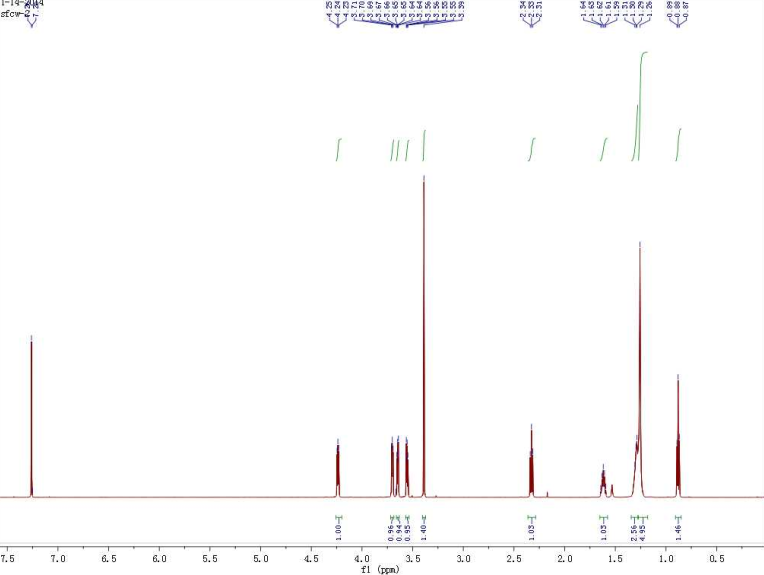


Fig.S1 ^1^H NMR spectrum of DGMEML

Table S1 ^1^H NMR spectroscopic data showing chemical composition of DGMEML

| Peak/no. | Peak area/region | Chemical structure |
| --- | --- | --- |
| 1 | 0.96 ppm | -CH_3_ |
| 2 | 1.29-2.25 ppm | -CH_2_ |
| 3 | 3.24 ppm | -OCH_3_ |
| 4 | 3.54-4.25 ppm | -CH_2_CH_2_O- |


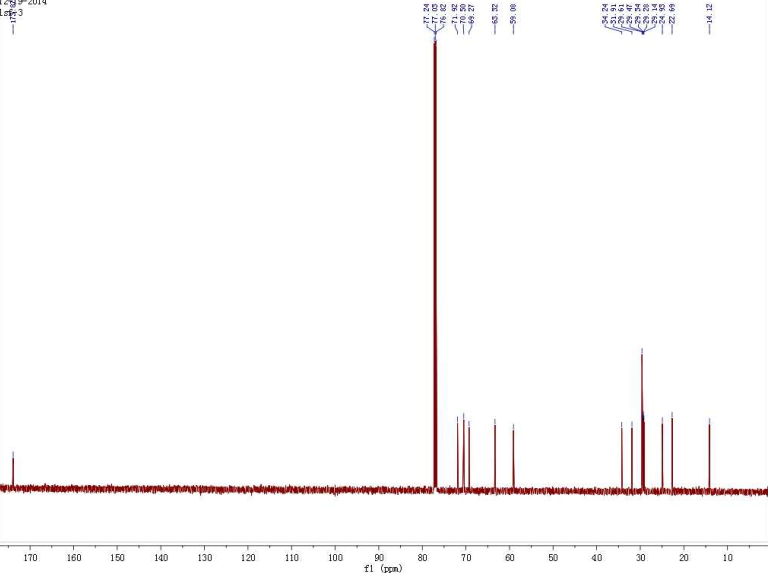


Fig.S2 ^13^C NMR spectrum of DGMEML

Table S2 ^13^C NMR spectroscopic data showing chemical composition of DGMEML

| Peak/no. | Peak area/region | Chemical structure |
| --- | --- | --- |
| 1 | 14.0 ppm | -CH_3_ |
| 2 | 23.1-33.6 ppm | -CH_2_ |
| 3 | 58.9 ppm | -OCH_3_ |
| 4 | 67.4-73.1 ppm | -CH_2_CH_2_O- |
| 5 | 172 ppm | -COOCH_2_- |

Fig.S3 FT-IR spectrum of DGMEML

Table S3 FT-IR data presenting various functional groups in DGMEML.

| Peak/no. | Wavenumber(cm^-1^) | Group attribution | Vibration tape |
| --- | --- | --- | --- |
| 1 | 2927 | -CH_2_-,-CH_3_ | asymmetric stretching |
| 2 | 2858 | -CH_2_-,-CH_3_ | symmetric stretching |
| 3 | 1743 | -C=O | stretching |
| 4 | 1458 | -CH_2_ | asymmetric bending |
| 5 | 1178 | C-O-C | asymmetric stretching |
| 6 | 1054 | C-O-C | symmetric stretching |
